# Genome sequencing of a novel *Verticillium dahliae* strain (huangweibingjun)

**DOI:** 10.1038/s41598-025-99279-z

**Published:** 2025-04-30

**Authors:** Vincent Ninkuu, Zhixin Liu, Hao Liu, Chunyang Li, Yaping Zhou, Qianli Zhao, Aizhi Qin, Mengfan Li, Peibo Gao, Lulu Yan, Xiao Song, Luyao Kong, Yajie Xie, Enzhi Guo, Xuwu Sun

**Affiliations:** State Key Laboratory of Cotton Bio-breeding and Integrated Utilization, State Key Laboratory of Crop Stress Adaptation and Improvement, Key Laboratory of Plant Stress Biology, School of Life Sciences, Henan University, 85 Minglun Street, Kaifeng, 475001 China

**Keywords:** Microbial genome, Pathogenic microbes, Secretory protein, Fungi secondary metabolites, Immunology, Microbiology, Physiology, Plant sciences

## Abstract

*Verticillium dahliae* is a soilborne pathogenic fungus that causes vascular discoloration and wilting in a broad spectrum of plant hosts, affecting about 400 species, such as cotton, potatoes, watermelon, cucumber, spinach, etc. In 2021, *V. dahliae* was estimated to cause about 15–20% reduction in cotton in China. Here, we report the genome sequencing of a novel strain named huangweibingjun, isolated from diseased cotton roots in the Henan province of China. The huangweibingjun genome consists of a total size of 35.84 Mb, GC content of 59.835%, and harbors six chromosomes (scaffold7561, scaffold7329, scaffold7795, scaffold5491, scaffold5473, and scaffold4511). The genome architecture showed a high diversity of cell wall-degrading secretory proteins that might influence the pathogenicity of the fungal strain. Moreover, preliminary metabolic pathway prediction showed that this novel strain synthesizes polyketide, terpenoids, shikimic acid-derived compounds and could also be aflatoxigenic. Consistent with other pathogenic microbes, the huangweibingjun genome comprises several virulent-associated genes. This genome assembly lays the foundation for further investigation of the pathogenicity of huangweibingjun.

**Supplementary Information:**

The online version contains supplementary material available at 10.1038/s41598-025-99279-z.

## Introduction

Cotton is an essential plant cultivated globally for its fiber as raw materials for fabric production, seeds for feed and food supplements, and straw for bioenergy generation^1–4^. Nevertheless, cotton production is constrained by disease-causing microbes, including fungi, which are estimated to exert approximately 15–20% yield loss annually^5–7^. Some fungal diseases of cotton include *Fusarium* and *Verticillium *wilt^8^, *Alternaria *leaf spot (foliar disease)^9^, and boll rot caused by *Corynespora cassiicola*^10^. Foliar-related infections form approximately 80–90% of cotton diseases^9,11^.

*Verticillium* (genus) *dahliae*(species) belonging to the phylum Ascomycota, class Sordariomycetes, and family Plectosphaerellaceae is an established member of the filamentous fungi genera classified by Nees von Esenbeck in 1817^12^. *V. albo-atrum *was the first pathogenic strain isolated in 1879 from potatoes in Germany^12^. The diversity of *Verticillium* has now expanded, forming several groups, including saprophytes, plants, and animal disease-causing *Verticillium *spp^13^. Current DNA sequencing and phylogenetic tools have accelerated the discovery of the evolutionary histories of these species and resolved several outstanding controversies in their identifications^14^. Apart from cotton and potato plants, *V. dahlia *has a broad range of hosts, such as watermelon, tomato, strawberry, lettuce, eggplant, chilli pepper, and cabbage^12,15^. The symptoms accompanying *V. dahliae*infections include leaf wilting, plant dysplasia, vascular bundle browning, yellowing, and early death^16^. These symptoms highlight the economic importance of the pathogen.

Several whole-genome sequencing studies of diverse strains of *V. dahliae* with varying genome sizes, scaffold lengths, and effector proteins have been identified. The defoliating and nondefoliating strains of *V. dahliae*XJ592 and XJ511 showed varied genome characteristics, such as 35 and 34 million each of genome sizes, respectively. The GC contents in the XJ592 and XJ511 strains also exhibited slight variations of 35.28% and 53.98%, respectively^17^. The secretory proteome was predicted to be 793 in XJ592 and 794 in XJ511, indicating the closeness of these strains. Identifying these proteins is significant in designing appropriate control factors against the diseases they cause. For example, *Verticillium dahliae* SnodProt1-Like Protein, VdCP1 expression increases throughout the infection process of *V. dahliae*, and its mutant lines showed no effect on pathogenesis^18^.

Different fungi strain harbor varying pathogenicity effects and can interact differently with host immunity^19^. These variations can be elucidated at the genomic level via genome sequencing analysis. Here, we report the genome-sequencing of a novel *V. dahliae* strain, herein referred to as huangweibingjun, isolated from diseased cotton roots. We showed the genome architecture, predicted proteome, and virulence factors governing the pathogenicity of huangweibingjun.

## Results

### Morphological and evolutionary analysis of Huangweibingjun

**Fig. 1:**
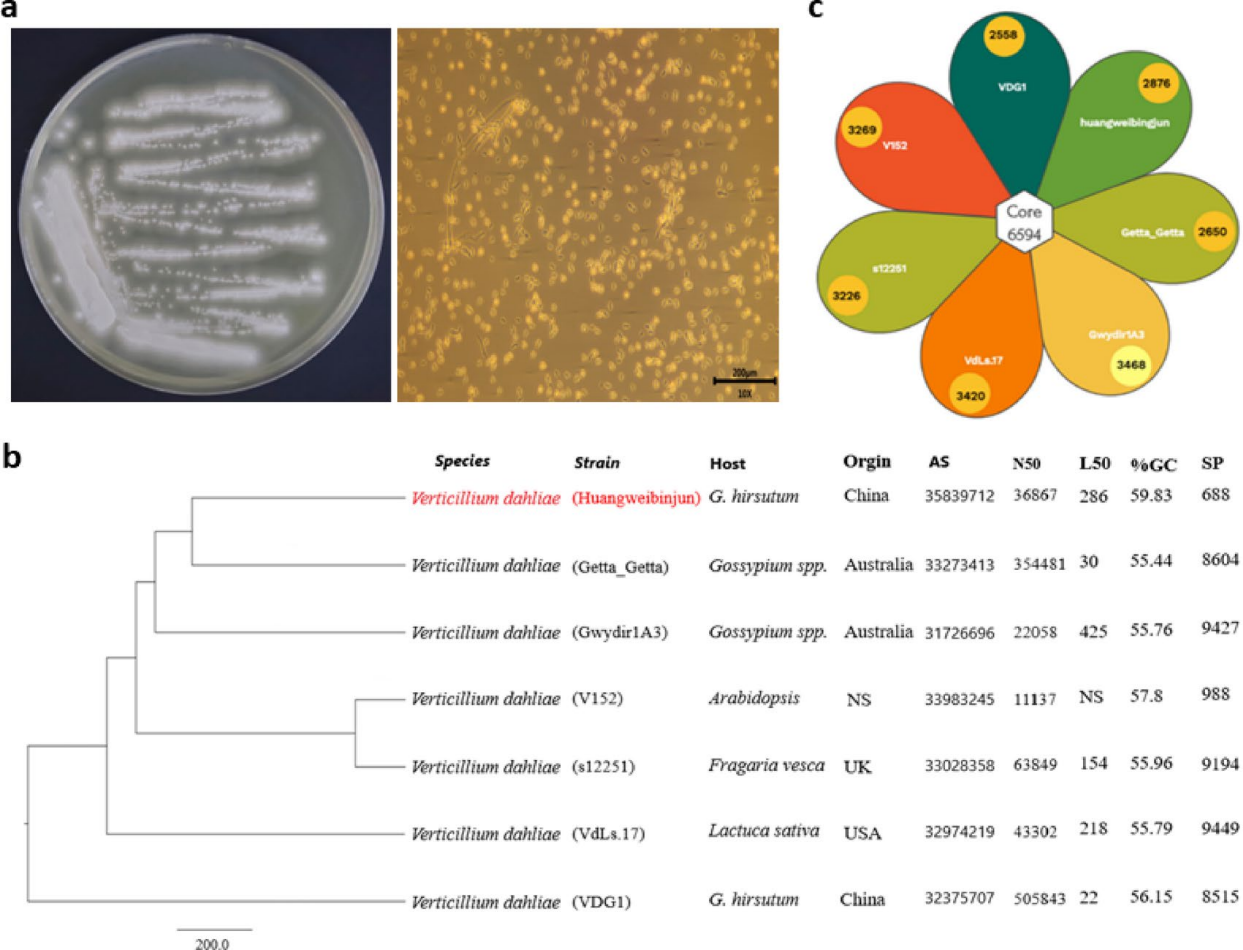
Morphological and molecular characterization of huangweibingjun strains. **(a)** Macro- and microscopic features of *Verticillium. dahliae* (huangweibingjun) grown on potato Dextrose Agar **(b)** ML phylogenetic tree characterizing huangweibingjun strain compared to other *Verticillium dahliae* strains. The red font shows the target strain, while black fonts are the strains that share a close identity with huangweibingjun. The origin of strain in the tree is also shown along a comparison of the Assembly size (AS), N50, L50, GC percentage, and the secreted proteome (SP) count **(c)** A petal diagram illustrating the shared genes among the seven strains. The core denotes the total number of common genes shared among these fungal strains.

Naturally infected root samples of cotton plants exhibiting wilting symptoms were collected from the field, surface-sterilized, inoculated on potato dextrose agar (PDA), and incubated in darkness. Observations of the *huangweibingjun* strain under a stereomicroscope (MZ16 F, Leica Microsystems, Germany) revealed fluffy, creamy white mycelia densely surrounding the inoculated roots and gradually spreading (Fig. 1a). Numerous oval conidia were produced, appearing detached from the conidiophores (Fig. 1a). Further evolutionary analysis (Maximum Likelihood phylogenetic tree) (Fig. 1b) of the protein sequences of *huangweibingjun* after genome sequencing showed that it is closely related to the getta_getta strain of *V. dahliae* but distantly related to the Gwydir1 A3, V152, s12251, and the VdLs.17 (Fig. 1b). A petal map of the pan and core genes shared among the reference and assembled genomes showed that 6,594 genes were shared among the seven species (Fig. 1c).

### Genome sequencing and assembly of the *V. dahliae* (huangweibingjun)

The huangweibingjun genome sequencing generated 57.0 Mb raw reads. After quality control analysis, 47.25 Mb clean reads were obtained. The genome comprises six chromosomes (scaffold7561, scaffold7329, scaffold7795, scaffold5491, scaffold5473, and scaffold4511). The start and end positions of each chromosome are shown in Table S1. The assembly statistics also showed that 10,328 scaffolds were ≥ 0 bp, 3228 scaffolds ≥ 500 bp, and 2293 scaffolds ≥ 1 kbp. The longest scaffold was 177,267 bp long. The N50 length (36,867) of huangweibingjun is shorter than its closest neighbor, Getta_Getta (35, 4481), but longer than Gwydir1 A3 (22058) and VdLs.17 (43302). However, the L50 length of huangweibingjun (286) is longer than the Getta_Getta (30) and VdLs.17 (218) strains. Additionally, the GC content (59.85%) of huangweibingjun is higher compared to the Getta_Getta (55.44), Gwydir1 A3 (55.76), s12251 (55.96), VdLa.17 (55.79), and VDG1 (56.15) (Table 1; Fig. 1c). We further assessed the completeness of the assembly, using Illumina reads and BUSCO, achieving approximately 97.57% of Illumina reads mapping to the new assembly and a high level of completeness of 96.12% in the BUSCO analysis.

**Table 1:** Genome features of *Verticillium dahliae* (huangweibingjun).

	Feature	*V. dahliae*
		(huangweibingjun)
Genome feature	Predicted genome size (bp)	35,839,712
	Number of genes (bp)	9901
	Total gene length (bp)	14,120,809
	Average gene length (bp)	1426.2
	GC content (%)	59.83
	Repeat rate (%)	21.18
	Gene length/Genome (%)	39.4
Reads feature	Total raw reads	57.00 M
	Total raw bases	8.55G
	Total raw > Q30	83.05%
	Total clean reads	47.25 M
	Total clean bases	7.01G
	Total clean > Q30	85.53%
	Total valid bases	81.96%
Assemble feature	Duplicate rate	18.04%
	Scaffolds number (≥ 0 bp)	10,328
	Scaffolds number (≥ 500 bp)	3228
	Scaffolds number (≥ 1 Kbp)	2293
	Longest scaffold (bp)	177,267
	N50 (bp)	36,867
	N75 (bp)	16,562
	L50 (bp)	286
	L75 (bp)	645
	Number of bases per 100kbp	1216.71

Total raw > Q30 is the number of bases greater than Q30 in the raw bases. Each sequence is sorted following its length, with values exceeding the initial 50% of the entire length named N50 and N75 for values exceeding 75%. L50 and L75 denote the number of contigs obtained at the cumulative length of N50 and N75.

### Gene prediction and functional annotation

Following the genome sequencing of *V. dahliae*, the GeneMark-ES (v4.33) software^20^ was used to predict 9,901 genes with a total length of 14,120,809 bp and an average gene length of 1,426.2 bp (Table 1). Non-coding RNAs (ncRNAs), which do not carry translatable information but are biologically significant in several other activities, were also annotated. 93% of these ncRNAs were ribosome-bound (rRNA), 7% were sRNA, but 0% were detected for tRNA (Supplementary Fig. S1). The sequence repeats in our *V. dahliae*genome, which might result in non-specific gene hits, was masked using the RepeatMasker before gene annotation^21^. Furthermore, tandem and interspersed repeats, including microsatellite sequences, satellite DNA, and small satellite sequences, are illustrated (Table S2).

#### Common functional annotation of genes

**Fig. 2:**
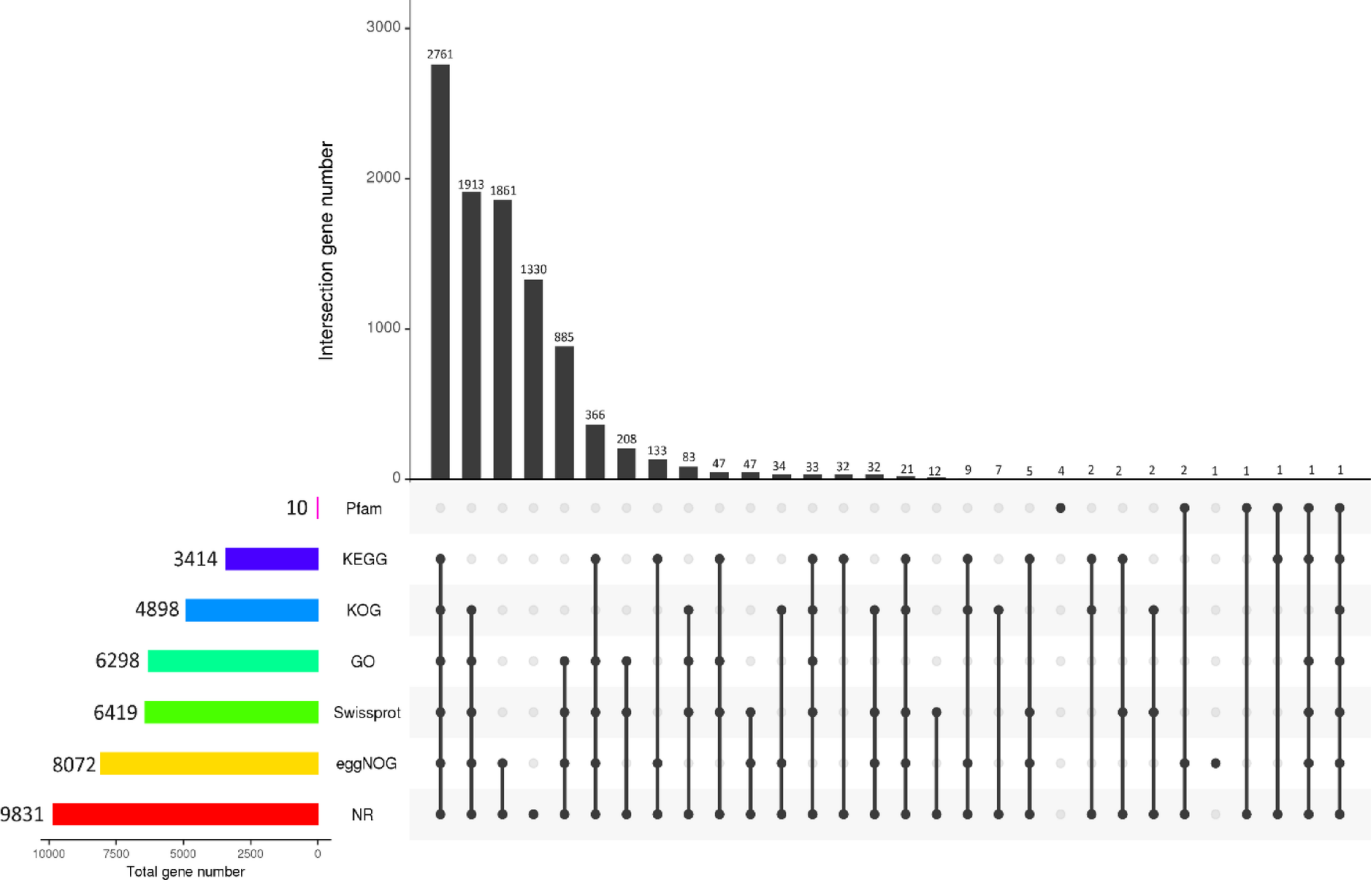
Upset plot illustrating common genes among various gene annotation analyses, including Pfam, KEGG, KOG, GO, Swissprot, eggNOG, and NR. The number of genes annotated in each database is shown at the end of each color bar corresponding to the database. The number of common annotated genes is also illustrated at the top of each.

The predicted genes were subjected to functional annotations using various tools. Among the 9,901 proteins in the *V. dahliae* genome, 9,831 proteins, representing 99.29%, were annotated on the non-redundant database (NR), while 6,419, representing 64.83%, were annotated on the Swissprot database. The KEGG database also annotated 3,414 (34.48%), KOG 4,898 (49.47%), and 8,072 (81.53%) proteins were annotated on the eggNOG. In addition, 6,298, representing 63.61%, were annotated on the GO database, while only ten (10) genes (0.10%) were annotated on Pfam (Table S3). The annotation of genes also showed that 2,761 genes were common among KEGG, KOG, GO, Swissprot, eggNOG, and NR, while 1,913 genes intersected among KOG, GO, Swissprot, eggNOG, and NR (Fig. 2). The distribution of annotated genes of the top ten species was also performed to determine the species huangweibingjun strain conform with. The results showed that huangweibingjun shared 54.98% gene similarity with *V*. *dahliae* VdLs.17, 38.99% with *V*. *longisporum*, and 4.57% with *V*. *alfalfae* VaMs (Supplementary Fig. S2).

#### EggNoG and gene ontology (GO) annotation of genes

**Fig. 3:**
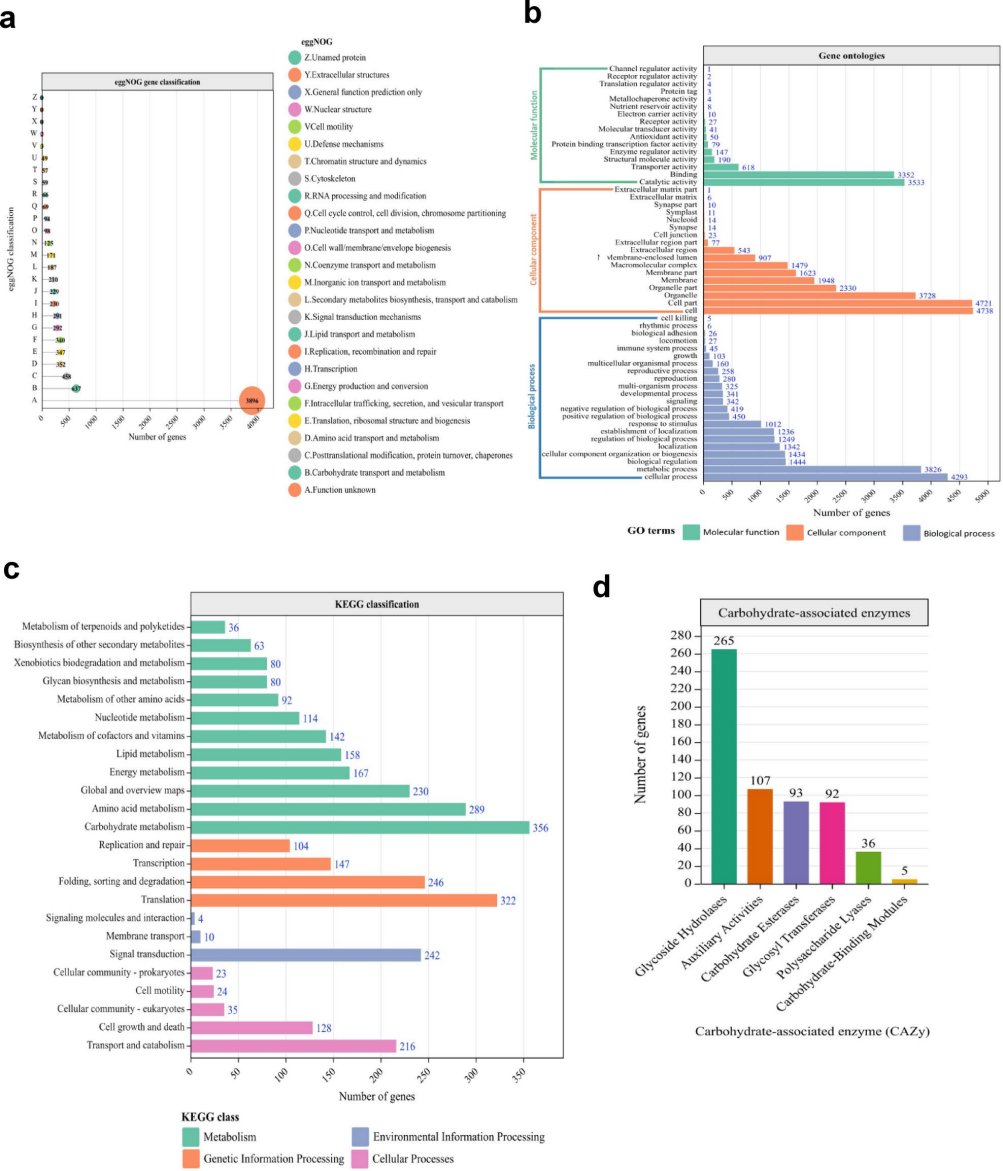
Functional annotation of predicted genes. **(a)** eggNOG classification of *V. dahliae* genes. The number of genes under each class is shown in the bubble plots. The vertical axis denotes the number of genes, while the horizontal axis represents eggNOG-classified genes. The interpretation of the alphabetical labeling is shown in the legends. (**b)** Gene ontology classification of predicted genes in the huangweibingjun genome. The x-axis represents the number of genes, and the y-axis represents the GO terms. Green bars represent molecular function, pink bars denote cellular components, and dark blue bars represent biological processes. The number of enriched genes is illustrated at the end of each bar. (**c)** KEGG annotation of huangweibingjun predicted genes. The vertical axis represents the enriched KEGG pathways, and the horizontal axis represents the number of enriched genes in each pathway, as illustrated at the end of each bar. Predicted genes enriched under KEGG are categorized into metabolism (green bars), genetic information processing (pink bars), environmental information processing (dark blue bars), and cellular process (magenta bars). (**d)** Classification of carbohydrate-associated enzyme (CaZy) in huangweibingjun genome. The vertical axis represents the number of annotated genes, while the horizontal axis denotes the annotated CaZy family proteins in the huangweibingjun genome. The number of genes in each CaZy family is shown at the top of each bar.

A quick view of the functional annotation of gene sequences was studied using the eggNOG mapper. The A-Z evolutionary genealogy of genes (Fig. 3a) illustrates the number of genes annotated under each category. A total of 8,262 genes were annotated. Out of these, 637 genes were annotated for carbohydrate transport and metabolism, 458 genes were identified to be responsible for the posttranslational modification, protein turnover, and chaperones, and no gene target was found among the query for extracellular structures, but 3,896 were annotated as genes with unknown functions (Fig. 3a). The GO annotation was also performed to identify the genes enriched under each ontology term: molecular functions, cellular components, and biological processes. The GO annotation (Fig. 3b) showed that 1,444 genes were enriched under biological regulation, five (5) genes under cell killing, 1,236 were enriched for establishing localization, and 342 genes were annotated under signaling. In addition, 79 protein-binding transcription factors were enriched under molecular function, while 27 genes were enriched under receptor activity (Fig. 3b). The GO annotations revealed several virulence-related genes.

#### Kyoto encyclopedia of genes and genomes (KEGG) annotation

The predicted genes were also subjected to the KEGG annotation to identify the metabolic pathways the genes encode. A total of 3,308 genes were annotated into 24 pathways. Among these include carbohydrate metabolism (356 genes), xenobiotic biodegradation and metabolism (80 genes), and glycan biosynthesis and metabolism (80 genes). Fungi secondary metabolites (63 genes) and metabolism of terpenoids and polyketides (36 genes) pathways were also enriched (Fig. 3c). The enriched pathways highlight the potential for successful environmental adaptation and pathogenicity of huangweibingjun.

#### The carbohydrate-associated enzyme (CaZy)

Carbohydrate-active enzymes (CaZy) break down complex carbohydrates and are responsible for phytopathogenic fungi nutrient acquisition from plant hosts, infection, and colonizing in the host^22,23^. The carbohydrate-active enzymes database was inferred for the CaZy annotation^24^ (Fig. 3d). The CaZy database constitutes over 300 protein families, including glycoside hydrolases (GHs), which comprise almost half of the CaZy protein families in genomes and are crucial for the hydrolysis and/or transglycosylation of glycosidic bonds. In the huangweibingjun genome, 265 members were annotated, making GHs the most abundant (44.3%) CaZy in the genome. The glycosyltransferases (GTs) catalyze the formation of glycosidic bonds from phospho-active sugar donors^25–27^. The *V. dahliae*genome harbors 92 members of GTs. In addition, thirty-six (36) polysaccharide lyases (PLs) members are present, forming 6% of the CaZy family in the huangweibingjun genome and are responsible for the cleavage of uronic acid-containing glycosidic bond^28^. The carbohydrate esterases (CEs) enhance GHs activities by eliminating ester-based modifications in mono-, oligo-, and poly-saccharides carbohydrates. Ninety-three (93) members of CEs are present in the *V. dahliae* genome, forming 15.5% of the CaZy members in the fungus. Carbohydrate-binding modules (CBMs) and the auxiliary activities comprise 5 and 107 members in the *V. dahliae* genome, respectively (Fig. 3d).

### Fungal pathogen virulence factors predicted in *V. dahliae* (huangweibingjun)

Virulence factors (VF) centrally regulate the interactions between plants and fungi during infection^29,30^. VFs are mostly chemical signals pathogens generate to enhance colonization in their host (biotrophic stage) or damage the host’s immune responses (necrotrophic stage). To determine the potential virulence factors in *V. dahliae*, protein sequences were queried on the Database of Fungal Virulence Factors (DFVF)^31^, and 1,057 proteins were predicted to be involved in huangweibingjun pathogenicity (Table S4). Compared to the closest species, *V. dahliae* (XJ592) genome contains 10,305 VF, and *V. dahliae*(XJ511) harbors 10,462 VF proteins^17^. Fifty-seven (57) VF proteins in huangweibingjun share ≥ 70% similarity to several virulence factors involved in various disease proliferation (Table 2). For example, 8647_t protein encodes a virulence factor (Q96 VA7_FUSOX) with 100% sequences consensus with the virulence factor in *Fusarium oxysporum* and involved in blight disease. In addition, 6038_t encodes a virulence protein belonging to the small GTPase superfamily Rho family and is 94.5% similar to the *M. oryzae* virulence factor responsible for causing rice blast infection. We found that 6074_t is 90.87% identical to the leaf spot-inducing protein harbored by *Colletotrichum lindemuthianum* (Table 2). These results indicate that huangweibingjun has high virulence factors similar to those already reported by VF, which could enhance the proliferation of several plant diseases.

**Table 2:** Verticillium dahliae (huangweibingjun) virulent factors with ≥ 70% similarity index.

Gene_ID	DFVF ID	Identity (%)	Organism	Description	Disease Key
9238_t	Q65YU2_FUSOX	87.89	*Fusarium oxysporum*	Unknown	Blight
9388_t	A4QV79_MAGO7	82.22	*Magnaporthe oryzae*	Unknown	Rice Blast
9173_t	Q9 C1 C6_COCCA	70.21	*Cochliobolus carbonum*	Unknown	Leaf Spot
8647_t	Q96 VA7_FUSOX	100	*Fusarium oxysporum*	Unknown	Blight
8338_t	O94101_BOTFU	82.75	*Botryotinia fuckeliana*	Unknown	Gray Mould
8147_t	Q9 C1U1_MAGGR	77.14	*Magnaporthe grisea*	Contains 1 histidine kinase domain	Leaf Spot
8300_t	Q5YCX0_FUSOX	71.55	*Fusarium oxysporum*	Unknown	Blight
7442_t	Q6XPX0_FUSOX	97.21	*Fusarium oxysporum*	Unknown	Blight
7569_t	Q2Q466_MAGGR	76.47	*Magnaporthe grisea*	Unknown	Leaf Spot
5225_t	Q96UH9_MAGGR	70.92	*Magnaporthe grisea*	Catalyzes ATP + a protein = ADP + a phosphoprotein.	Leaf Spot
5436_t	Q8 J2 N0_FUSOX	76.24	*Fusarium oxysporum*	Unknown	Blight
5441_t	Q9UQZ1_9PEZI	80.39	*Glomerella cingulata*	Unknown	Anthracnose
5811_t	CUTI_FUSSC	71.67	*Fusarium solani*	Hydrolysis cuticular barrier to fungi infection	Rot
5831_t	Q32 WF7_PHAND	71.97	*Phaeosphaeria nodorum*	Unknown	Glume Blotch
6038_t	Q2 KG56_MAGO7	94.5	*Magnaporthe oryzae*	GTPase superfamily Rho family	Rice Blast
6074_t	Q96 VL3_COLLN	90.87	*Colletotrichum lindemuthianum*	GTPase superfamily. Rab family.	Leaf Spot
6095_t	Q8 TFN6_COLGR	79.73	*Colletotrichum graminicola*	Unknown	Leaf Spot
6218_t	Q2 V086_GLOLA	86.85	*Glomerella lagenarium*	Unknown	Anthracnose
6230_t	A0SXQ6_MAGGR	83.25	*Magnaporthe grisea*	GTPase superfamily Rho family	Leaf Spot
6394_t	Q2PBY8_CLAPU	90.16	*Claviceps purpurea*	GTPase superfamily Rho family	Ergotism
91_t	Q00845_NECHA	78.21	*Nectria haematococca*	Unknown	Fruit Rot
121_t	Q9P304_CLAFU	73.83	*Cladosporium fulvum*	Cofactor: FAD (By similarity).	Leaf Mold
344_t	Q00845_NECHA	73.73	*Nectria haematococca*	Unknown	Fruit Rot
717_t	A6 N6 J8_FUSOX	72.88	*Fusarium oxysporum*	Contains 1 Zn(2)-C6 fungal-type DNA-binding domain	Blight
913_t	Q8 TFN4_COLGR	79.88	*Colletotrichum graminicola*	Unknown	Leaf Spot
949_t	Q9UW03_BOTFU	70.37	*Botryotinia fuckeliana*	Belongs to the ABC transporter superfamily	Gray Mould
1228_t	Q5 J4D6_PHAND	76.71	*Phaeosphaeria nodorum*	Catalyzes Acetyl-CoA + H(2)O + glyoxylate = (S)-malate + CoA.	Glume Blotch
1432_t	Q8 TGD1_FUSOX	81.35	*Fusarium oxysporum*	Member of mitochondrial carrier family.	Blight
1841_t	Q8 J286_COLLN	78.52	*Colletotrichum lindemuthianum*	Member of cation transport ATPase (P-type) family.	Leaf Spot
1860_t	A4R2P0_MAGO7	80.36	*Magnaporthe oryzae*	Contains 1 SH3 domain	Rice Blast
2056_t	Q96 WW9_GIBPU	72.75	*Gibberella pulicaris*	Mmber of the ABC transporter superfamily.	Rot
2084_t	Q8 NK75_GLOLA	80.88	*Glomerella lagenarium*	Unknown	Anthracnose
2162_t	Q8 TFN5_COLGR	85.75	*Colletotrichum graminicola*	Unknown	Leaf Spot
2365_t	TREB_MAGO7	76.51	*Magnaporthe oryzae*	Enhances pathogenicity and rice blast hyphae invasion	Rice Blast
2537_t	O13316_MAGGR	82.25	*Magnaporthe grisea*	Unknown	Leaf Spot
2574_t	Q86ZP4_GIBZA	79.15	*Gibberella zeae*	Unknown	Seedling Blight
2660_t	Q875L7_MAGGR	83.02	*Magnaporthe grisea*	Unknown	Leaf Spot
2715_t	Q2L7 J5_FUSSO	80.45	*Fusarium solani*	Unknown	Rot
2825_t	Q0 WXM3_FUSOX	79.41	*Fusarium oxysporum*	Contains 1 Zn(2)-C6 fungal-type DNA-binding domain	Blight
2831_t	A8QJI7_FUSOX	90.12	*Fusarium oxysporum*	Contains 1 Zn(2)-C6 fungal-type DNA-binding domain	Blight
2979_t	D3YIG6_SPOSC	88.81	*Sporothrix schenckii*	Unknown	Rose-picker’s Disease
3226_t	Q874 F3_MAGGR	71.05	*Magnaporthe grisea*	ABC transporter superfamily	Leaf Spot
3268_t	Q5 K2R7_CLAPU	79.91	*Claviceps purpurea*	Catalyzes ATP + a protein = ADP + a phosphoprotein.	Ergotism
3455_t	A4RKI4_MAGO7	85.84	*Magnaporthe oryzae*	Unknown	Rice Blast
3483_t	A4R1 F0_MAGO7	77.05	*Magnaporthe oryzae*	Unknown	Rice Blast
3484_t	A4R1 F0_MAGO7	78.63	*Magnaporthe oryzae*	Unknown	Rice Blast
3529_t	Q99324_SEPLY	74.63	*Septoria lycopersici*	Unknown	Leaf Spot
3553_t	A6ZIB8_MAGGR	87.3	*Magnaporthe grisea*	Contains 1 FAD-binding FR-type domain.	Leaf Spot
4014_t	Q00350_COCCA	77.78	*Cochliobolus carbonum*	Endohydrolysis of (1->4)-beta-D-xylosidic linkages in xylans.	Leaf Spot
4104_t	P79068_GLOLA	75.87	*Glomerella lagenarium*	Unknown	Anthracnose
4115_t	O93802_ALTAL	76.06	*Alternaria alternata*	SDR family.	Leaf Spot
4241_t	A4RGG9_MAGO7	76.26	*Magnaporthe oryzae*	SDR family.	Rice Blast
4253_t	A9Z1 V6_PHAND	73.25	*Phaeosphaeria nodorum*	Unknown	Glume Blotch
4367_t	A4R2 V5_MAGO7	80.84	*Magnaporthe oryzae*	Retention and specificity of the LER protein and normal vesicular traffic through the Golgi bodies.	Rice Blast
4504_t	Q6 WP53_BOTFU	78.65	*Botryotinia fuckeliana*	PPIases accelerate protein folding	Gray Mould
4526_t	Q8 × 125_GLOLA	93.3	*Glomerella lagenarium*	ATP + a protein = ADP + a phosphoprotein.	Anthracnose
6941_t	A4RFD4_MAGO7	86.53	*Magnaporthe oryzae*	Catalyzes S-adenosylmethionine formation from methionine and ATP	Rice Blast

LER proteins: luminal endoplasmic reticulum proteins; SDR: short-chain dehydrogenases/reductases.

**Fig. 4:**
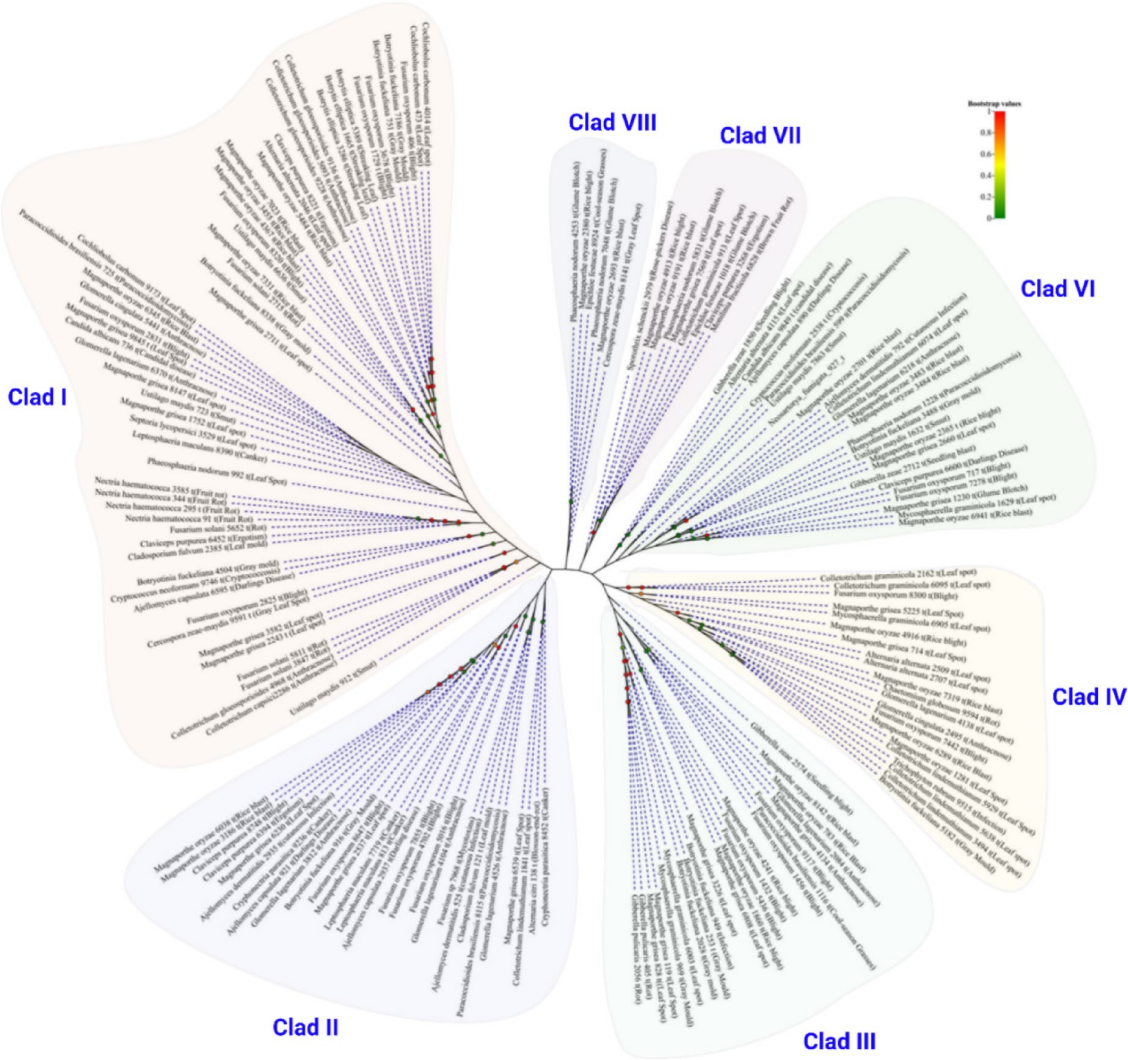
Evolutionarily analysis of *Verticillium dahliae* virulent factors (≥ 50% similarity). Protein sequences of *Verticillium dahliae* virulence factors were clustered on MEGA 11, and the evolutionary tree was constructed using the ML method.

Furthermore, we analyzed the evolutionary antecedence of the predicted virulence proteins with ≥ 50% sequence identities using a neighbor-joining phylogenetic tree. The evolutionary tree cladded the VF proteins into eight groups (I-VIII). Moreover, we found that some predicted VF in huangweibingjun are cladded together, indicating they may have evolved from a common ancestry. For example, the leaf spot virulence factor secreted by *Cochliobolus carboum*, blight virulence from *Fusarium oxysporum*, the streaking leaf virulence produced from *Botrytis elliptica*, and rice blast virulence from *Magnaporthe oryzae* are evolutionarily related in our newly isolated huangweibingjun (Clad I) (Fig. 4). Similarly, the rot-inducing protein elicited by *Gibberella publicaries*, the leaf spot proteins from *M. grisea* and *Mycospopharella graminicola*, and the blight virulence factor secreted by *M. grisea* might have evolved from a common ancestry (Clad III) (Fig. 4), suggesting their common virulence history might provide clues to engineering programs towards mitigating the disease they cause.

Pathogens interact with their potential host at the cellular, molecular, or organismal level to induce their virulence effect^32–34^. Several proteins are involved in these interactions to enhance the success of pathogens. Therefore, the pathogenicity predictive tool, the Pathogen-Host interaction (PHI) database^35^, was inferred to predict gene orthologs in huangweibingjun with increased virulence. 2,496 genes were annotated to be involved in PHI (Table S5), of which 33 genes were predicted to harbor a hypervirulence effect. Some notable PHIs factors are effector proteins, pectate lyase, and transcription factors (Table 3).

**Table 3:** Pathogen-host interaction (PHI) factors predicted in *V. dahliae* (huangweibingjun).

Gene_ID	Identity (%)	Protein_ID	PHI_ID	Gene name	Pathogen_species	Host_species	Function
9625_t	34.21	I1S914	PHI:1721	GzZC036	*Fusarium graminearum*	*Triticum spp.*	Transcription factor
9887_t	37.31	A0 A0D3LXG7	PHI:4620	PcPL20	*Phytophthora capsici*	*Capsicum annuum*	Pectate lyase
334_t	40.69	A0 A0D3LX64	PHI:4618	PcPL15	*Phytophthora capsici*	*Capsicum annuum*	Pectate lyase
766_t	64.65	K9Y567	PHI:4506	So (soft)	*Epichloe festucae*	*Festuca arundinacea*	Symbiotic
1019_t	31.53	Q09 JB7	PHI:2482	AlHK1	*Alternaria longipes*	*Nicotiana tabacum*	Group III two-component Histidine Kinase
1489_t	32.25	Q5 KEF4	PHI:4575	NTH2	*Cryptococcus neoformans*	*Mus musculus*	Trehalose Homeostasis
1597_t	39.55	A0 A0D3LX64	PHI:4618	PcPL15	*Phytophthora capsici*	*Capsicum annuum*	Pectate lyase
1806_t	39.34	G8 AA67	PHI:2476	CcpelA	*Colletotrichum coccodes*	*Solanum lycopersicum*	Pectate lyase
1939_t	43.4	G8 AA67	PHI:2476	CcpelA	*Colletotrichum coccodes*	*Solanum lycopersicum*	Pectate lyase
2111_t	26.24	I1R980	PHI:2393	OMST-like	*Fusarium graminearum*	*Triticum* spp.	No data found
2373_t	26.37	I1R980	PHI:2393	OMST-like	*Fusarium graminearum*	*Triticum* spp.	No data found
2379_t	9.74	A0 A0D3LX64	PHI:4618	PcPL15	*Phytophthora capsici*	*Capsicum annuum*	Pectate lyase
2921_t	42.5	G4MVX4	PHI:3216	MoCDIP4	*Magnaporthe oryzae*	*Nicotiana tabacum*	Effector (avirulence determinant)
3255_t	27.36	Q9 AT28	PHI:666	NPP1	*Phytophthora parasitica*	*Nicotiana tabacum*	Effector protein
3644_t	47.73	Q870 A3	PHI:315	PacC	*Fusarium oxysporum*	*Nicotiana tabacum*	pH signaling transcription factor
5093_t	71.08	G8 AA67	PHI:2476	CcpelA	*Colletotrichum coccodes*	*Nicotiana tabacum*	Pectate lyase
5520_t	25	I1R980	PHI:2393	OMST-like	*Fusarium graminearum*	*Nicotiana tabacum*	No data found
5872_t	24.94	Q87DK1	PHI:3663	PD0681	*Xylella fastidiosa*	*Nicotiana tabacum*	Glucose kinase, Glk
5967_t	57.4	G8 AA67	PHI:2476	CcpelA	*Colletotrichum coccodes*	*Nicotiana tabacum*	Pectate lyase
6349_t	33.62	D0 KML5	PHI:2735	RsmA	*Pectobacterium wasabiae*	*Nicotiana tabacum*	Posttranscriptional regulator
6518_t	56.42	G4MS28	PHI:4493	CGT	*Magnaporthe oryzae*	*Nicotiana tabacum*	Ceramide glucosyltransferase
6762_t	50.45	G8 AA67	PHI:2476	CcpelA	*Colletotrichum coccodes*	*Nicotiana tabacum*	Pectate lyase
7473_t	24.64	I1R980	PHI:2393	Related to OMST	*Fusarium graminearum*	*Nicotiana tabacum*	No data found
7483_t	44.91	I1R980	PHI:2393	OMST-like	*Fusarium graminearum*	*Nicotiana tabacum*	No data found
7820_t	25.78	Q87DK1	PHI:3663	PD0681	*Xylella fastidiosa*	*Nicotiana tabacum*	Glucose kinase, Glk
8034_t	81.46	I1RA06	PHI:6258	DHS	*Fusarium graminearum*	*Nicotiana tabacum*	Deoxyhypusine synthase
8502_t	65.23	A9QUB2	PHI:2403	CSN1	*Fusarium solani*	*Nicotiana tabacum*	Chitosanase
8775_t	30.51	I1R980	PHI:2393	OMST-like	*Fusarium graminearum*	*Nicotiana tabacum*	No data found
8807_t	31.62	G8 AA67	PHI:2476	CcpelA	*Colletotrichum coccodes*	*Nicotiana tabacum*	Pectate lyase
8901_t	32.85	G8 AA67	PHI:2476	CcpelA	*Colletotrichum coccodes*	*Nicotiana tabacum*	Pectate lyase
9117_t	63.17	Q870 A3	PHI:315	PacC	*Fusarium oxysporum*	*Nicotiana tabacum*	pH signaling transcription factor
9136_t	51.98	G8 AA67	PHI:2476	CcpelA	*Colletotrichum coccodes*	*Nicotiana tabacum*	Pectate lyase

*O*MST (*O*-methylsterigmatocystin oxidoreductase), P450 (cytochrome P450 monooxygenase).

List of Figures and legends.

### *V. dahlia*e (huangweibingjun) effectors and PAMPs-inducing proteins

Plant pathogens directly inject effector proteins into their host cells to induce host cell signaling or cause the suppression of the innate immune systems of their host^30,36,37^. However, plants have evolved intracellular or cell surface receptors for pathogens’ recognition through effector perception termed pathogen-associated molecular patterns (PAMPs)^37–40^. The PAMPs-inducing factors in huangweibingjun are highlighted below.

Fungi secretory proteins significantly contribute to their environmental adaptation and successful colonization in their target hosts^30,41,42^. These secretory proteins harbor domains that stringently involve carbohydrates and protein degradations such as pectate lyase, glycoside hydrolase, protease, etc^43,44^. For example, FoEG1, a glycoside hydrolase family 12 protein isolated from *Fusarium oxysporum*, induces apoplastic cell death and promotes infections in cotton and tomatoes^45^. The secretory proteome of huangweibingjun was analyzed using the signal peptide predictive tool (signalP, version 4.1)^46^, and 688 proteins were predicted (Table S6).

**Fig. 5:**
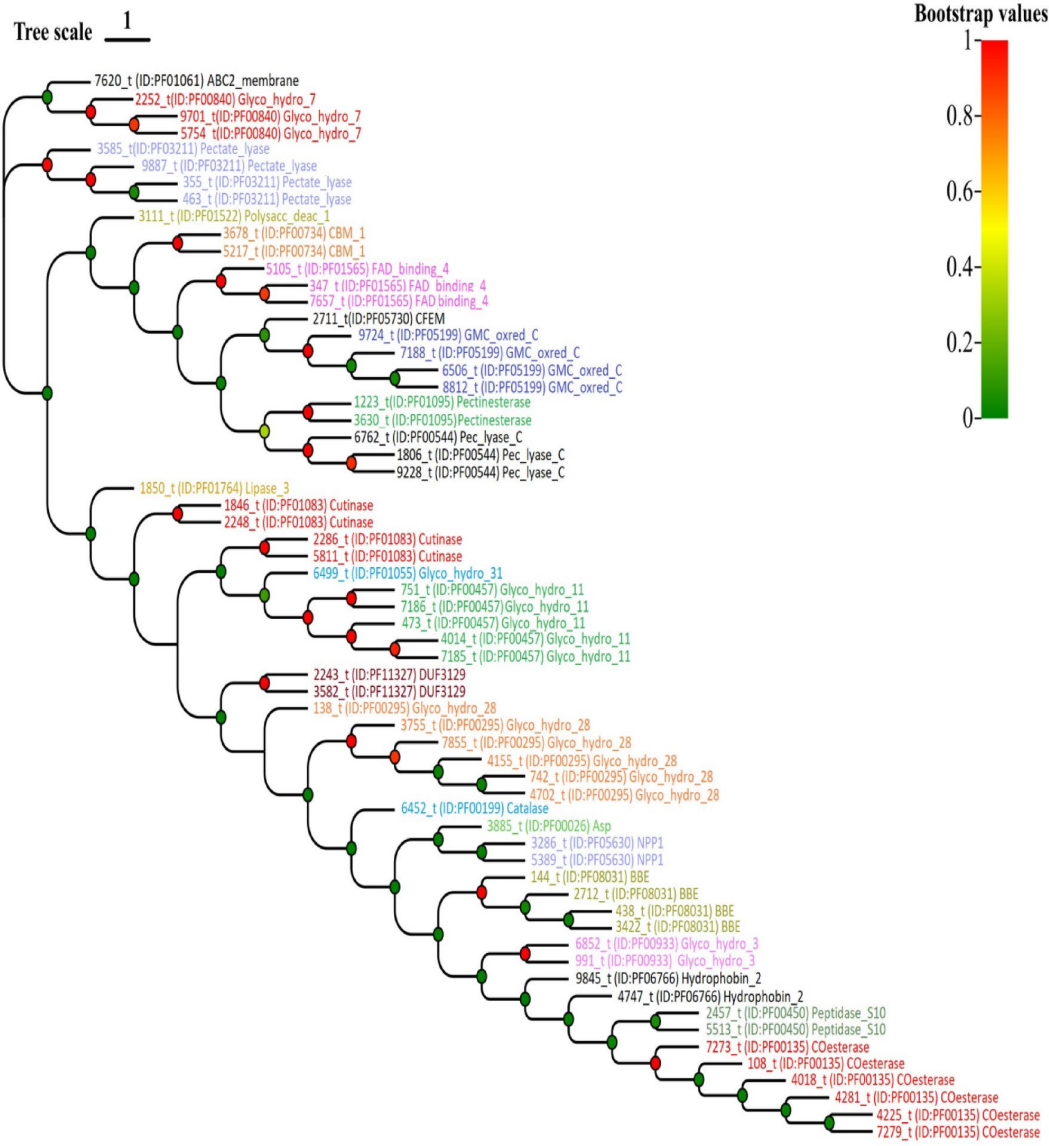
Phylogenetic classification of *Verticillium striae* secretory proteome. Each protein’s locus ID, Pfam ID, and short description are used on the leaf. E.g., 7620 t (locus ID) ID: PF01061(Pfam ID) ABC2 membrane (short description). The gradient shows the bootstrap scale.

These effector proteins share sequence similarity with other secreted proteins annotated in other pathogens. To further understand the classification of our huangweibingjun**-**specfic secreted effector proteins, the protein sequences were extracted and aligned using MUSCLE, and a neighbor-joining phylogenetic tree was constructed using MEGA 11 (Fig. 5). Four members of the pectate lysate genes involved in host tissues degrading and nutrient foraging for pathogen colonization were found among the effectors classified. The Glycoside hydrolase superfamily (Glyco_hydro) proteins are members of the CaZy enzyme family known for their involvement in the hydrolysis of glycosidic linkages mainly responsible for cell wall degradation to enhance pathogen colonization^47,48^. Based on amino acid sequence similarities, five families comprising 17 proteins classified as glycoside hydrolase 7, 10, 11, 28, and 31 were predicted in *V. dahliae*. A recent investigation demonstrated that VdCE11 promotes cotton susceptibility by increasing the hydrolase activity of GhAP1^49^. In addition, silencing the hydrophobin 2 protein, *VdHP1*, from *V. dahliae* stifled microsclerotia formation, enhanced hydrophilicity, and promoted *V*. *dahliae’s*sensitivity to NaCl. In contrast, the wild type induced cell death and promoted the pathogenicity of the fungi^50^. Our secreted proteome mining identified two homolog proteins encoding hydrophobin 2 (Fig. 5 and Tables S7 and S8).

### Secondary metabolic and mycotoxin pathways

Filamentous fungi are crucial producers of mycotoxins and other secondary metabolites, which perhaps aid their exploitation for niche by fighting off competitors. The metabolic pathways analysis of *V. dahliae* (huangweibingjun) showed a potential for aflatoxin biosynthesis as three proteins were identified to enrich the aflatoxin synthetic pathway (3244_t, 4106_t, 6737_t) (Supplementary Fig. S3a). Whereas 36 genes were predicted to be responsible for terpenoids and polyketides biosynthesis in huangweibingjun (Supplementary Fig.s S3b and S4), 23 genes were found in the genome of *M. grisea*^51^. Microbial niche exploitation has also been suggested to involve efficient detoxification of xenobiotic compounds to colonize a toxic environment by depending on carbon, sulfur, phosphorus, and nitrogen. A further prediction of genes showed that huangweibingjun could exhibit cosmopolitan characteristics as 144 genes were predicted to encode Xenobiotics biodegradation and metabolism.

Furthermore, glycine, serine, and threonine metabolism influence fungi growth and pathogenicity, and enhanced levels of these metabolites promote fatty acid biosynthesis and the TCA cycle for fungal nutrient provision^52^. Therefore, their potential metabolism in huangweibingjun could influence their adaptation to a broad spectrum of niches (Supplementary Fig. S5a). Cysteine and methionine are sulfur-containing amino acids crucial to protein synthesis and cell life. For instance, the metabolism of these metabolites in *Fusarium graminearum* is regulated by *FgMet3* and *FgMet14*for vegetative growth, sexual development, pigment formation, and penetrability in the host^53^. The success of pathogens relies on efficient reproduction. Therefore, the cysteine and methionine enrichment in huangweibingjun may influence its pathogenicity (Supplementary Fig. S5b).

## Discussion

Cotton production is constrained by several biotic factors^54,55^. In recent times, *V. dahliae*, arguably the most devastating pathogen in cotton production, was estimated to exert a 32.49% loss in cotton yield in China in 2021^56^. Although the genome of this fungus is sequenced, enhancing our understanding of its pathogenicity, the evolution of several strains harboring varying pathogenicity effects poses limitations to conquering this *V. dahliae *disease in plants. This study presents the genome characteristics of a novel strain, huangweibingjun, and its distinguishing features with its closer and distant neighbors. The secretory proteome of huangweibingjun is highly diverse and includes a protein family that aids in its pathogenesis. For example, four members (3, 7, 11, and 28) of the glycosyl hydrolase family proteins were identified in the huangweibingjun strain. Glycosyl hydrolase family 28, in particular, have been functionally elucidated as extracellular proteins that hydrolyze glycosidic bonds in pectin to enhance the delivery of the virulence factors of pathogenic fungi^57^. Additionally, VdGH7a, a member of GH7, exhibits hydrolytic activity against 1,4-β-glucan and induces cell death in *N. benthamiana* leaves^58^.

The cell walls of plants composed of cellulose, hemicellulose, pectin, etc., are the external protective barrier to foreign invasion^59^. Successful pathogens overcome this barrier by secreting cell wall-degrading enzymes such as pectate lyase, cutinases, pectinases, etc., to digest these carbohydrates^23^. As previously reported, all these proteins were predicted in the huangweibingjun genome, suggesting its potential pathogenicity^60^. Brito, et al.^61^ reported that Xyn11 (xylanase), a GH11 protein isolated from *Botrytis cinerea*, induces cell death in leaves and enhances the virulence of *B. cinerea.* In *V. dahliae*, two GH12 proteins, VdEG1 and VdEG3, were identified as cellulase-producing protein that triggers PAMPs, cause cell death, and upregulate immune responses in *N. benthamiana*^37,62^. Furthermore, VdPEL1, a pectate lyase, and VdCUT11, a cutinase, enhance the virulence of *V. dahliae *and activate PAMP activities in plants^63,64^.

The potential of huangweibingjun to elicit some secondary metabolites was also predicted. These secondary metabolites are categorized into terpenoids, polyketides, non-ribosomal peptides, and shikimic acid-derived compounds^65^. Whereas the biosynthesis of these metabolites was predicted in the huangweibingjun genome, previous findings suggest fungi secondary metabolites are responsive to stressors such as oxidative stress^66^. Mycosporines and polyol metabolites secreted by fungi are also pathogenicity factors and enhance fungi resistance against abiotic stresses, including UV and temperature^67^. Similar to *Aspergillus* and *Fusarium*spps., huangweibingjun could be a mycotoxin producer^68,69^, as three genes encoding aflatoxin biosynthesis were predicted.

The detailed genome characteristics of huangweibingjun provide a crucial foundation for future research on mitigating its impact and understanding its disease-causing effects in cotton plants.

## Materials and methods

### Isolation and identification of *V. dahliae* (huangweibingjun)

Naturally-infested cotton roots were sterilized in 75% ethanol for 10 min, rinse in distilled water 5 times and inoculated on potato dextrose agar plates for 48 h at 28 °C. Mycelia were harvested and subcultured on fresh PDA plates until pure cultures were obtained. Pure cultures were allowed to grow for seven days before they were microscopically observed using a light microscope. The identity of the isolate was determined through genomic DNA extraction using the Sangon DNA extraction kit and gene amplification of the Internal Transcribed Spacer (ITS) region, using primer pairs ITS5: GGAAGTAAAAGTCGTAACAAGG and ITS4: TCCTCCGCTTATTGATATGC, as described^70^.

### Evolutionary analysis of *V. dahliae* (huangweibingjun)

The evolutionary antecedent of huangweibingjun was inferred by aligning protein sequences with orthologous single-copy genes for all species. The ParaAT (version 20)^71^and the RAxML^72^ were used to construct the ML evolutionary tree based on the General Time Reversible (GTR) and the GAMMA-distributed model.

### Library construction and sequencing

The genomic DNA from huangweibingjun was extracted using a kit and protocol from Omega Fungal DNA Kit D3390-02. The genomic DNA was subjected to electrophoresis quantification. The quantified DNA samples were subsequently fragmented into 350 bp by Covaris, and the sequencing libraries were built using the TruSeq DNA LT Sample Prep kit. The DNA fragments underwent end repair, A-tailing, sequencing adapters, purification, PCR amplification., and finally, the library construction. After the library is qualified, the sequencer was used for double-end sequencing.

### Genome assembly

#### Contig assembly

The de Bruijn graph approach was used to assemble the genome based on the generated paired-end sequencing data^73,74^. Sequencing reads from all small-fragment libraries were trimmed into shorter sequences, and de Bruijn graphs were constructed based on their overlapping relationships. To simplify the de Bruijn graph, branches that were unexpendable or had low coverage, primarily due to sequencing errors, were removed. Read alignment information was used to resolve bifurcations caused by repeat regions, and a random selection strategy was employed to merge a limited number of heterozygous sites. Despite simplification, the de Bruijn graph retains numerous unresolved fork sites, prompting sequence fragmentation at each fork to generate the initial contigs^73,74^.

#### Scaffold assembly

The reads obtained from all library sequencing were aligned back to the preliminary contigs, and the contigs were assembled into scaffolds using the ligation relationship between the reads and the insert size information^73,74^.

#### Raw data processing and quality control

Quality control analysis on raw read was performed using the Trimmomatic software^75^. The sequencing data with low-quality reads, ambiguous bases, and sequence adaptors were removed. The clean reads obtained were used for further downstream analysis.

#### Estimation of genome size by K-mer method

The consistency of the sequenced genome size to that of the predicted genome sizes, repeat structure, heterozygous rate, and sequencing depth was predicted using the K-mer analysis^76^.

#### Gene prediction and functional annotation

The Illumina-generated data were subjected to downstream bioinformatics analysis. The fungi genome is rich in repetitive sequences and transposable elements; therefore, the GeneMark-ES (v4.33) tool was applied to predict the genes in the Huangweibingjun genome^20^. Depending on their classes, different predictive tools were also used to predict the non-coding RNA (ncRNA). For tRNA, the tRNAscan-SE (v1.3.1) tool was used^77^, while rRNA and sRNA in the huangweibingjun genome were predicted using RNAmmer (v1.2) and Rfam (v10.0) tools, respectively^78,79^. Further, we predicted the repetitive nucleotide sequences in the huangweibingjun genome using the RepeatMasker (v4.0.7) software^21^. The prophages in the genome of huangweibingjun were predicted using the PhiSpy (v2.3) software^80^.

The sources of sequence redundancy (HTGS, EST, GSS, STS) were removed using the non-redundant database (https://www.ncbi.nlm.nih.gov/). The SwissProt functional annotation was performed using the online tool http://www.uniprot.org/. Further, the Kyoto Encyclopedia of Genes and Genomes (KEGG) database (http://www.genome.jp/kegg/pathway.html) was also used to annotate the enriched pathway among the predicted genes in the huangweibingjun genome. The EuKaryotic Orthologous Groups, or KOGs, functional annotation of genes was performed using the online tool at https://www.creative-proteomics.com/services/kog-annotation-analysis-service.htm. To further understand the evolutionary histories of genes and their functions, the EggNOG annotation was performed using the online tool http://eggnog.embl.de/. The Gene Ontology annotation of genes classified predicted genes into three ontology terms, including biological processes, molecular functions, and cellular components, using the GO term finder.

The http://www.cazy.org/database was also used to analyze and classify the carbohydrate-associated enzymes in the huangweibingjun genome^24^. The annotation of the virulence factors harbored by huangweibingjun was also performed using the Fungal Pathogen Virulence Factors (DFVF) database^31^. In contrast, the signal peptide predictive tool SignalP (v4.1) was used to predict the secretory proteome^46^. In addition, the pathogen-host interaction factors were annotated using the Pathogen-host interaction (PHI) database^81^.

## Electronic supplementary material

Below is the link to the electronic supplementary material.

Supplementary Material 1

Supplementary Material 2

## Data Availability

Sequence data that support the findings of this study have been deposited in the China National Center for Bioinformation with the primary accession code CRA017486.

## Declarations

### Statement of permission for collecting Verticillium dahliae

Experimental research and field studies on plants (either cultivated or wild), including the collection of plant material have complied with relevant institutional, national, and international guidelines and legislation. The experimental studies and collection of *Verticillium dahliae* belongs to fungus, not plants.
