# Supplementary material for: Genome sequencing of a novel Verticillium dahliae strain (huangweibingjun)
Source: Sci Rep. 2025 Apr 30;15:15143. doi: 10.1038/s41598-025-99279-z (PMC12044074; doi:10.1038/s41598-025-99279-z)
Supplement: Supplementary file 1 — Supplementary Material 1 [file 41598_2025_99279_MOESM1_ESM.docx]

**Supplementary Figures**

**
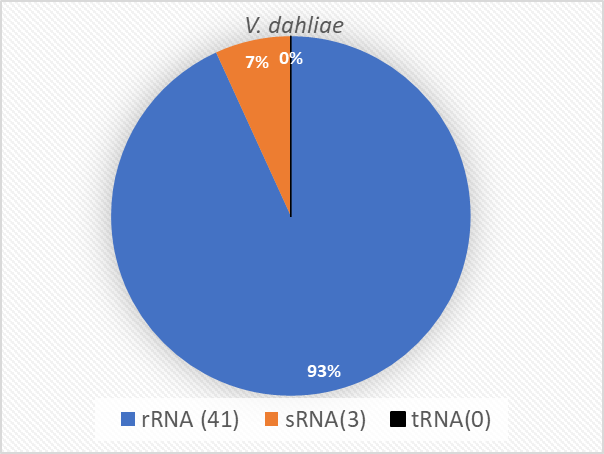
**

**Supplementary Fig. S1.** Non-coding RNAs (ncRNAs) in huangweibingjun illustrating the proportion of rRNA, sRNA, and tRNA

**Supplementary Fig. S2.** The distribution of annotated genes among the top ten species

**(a)**

**
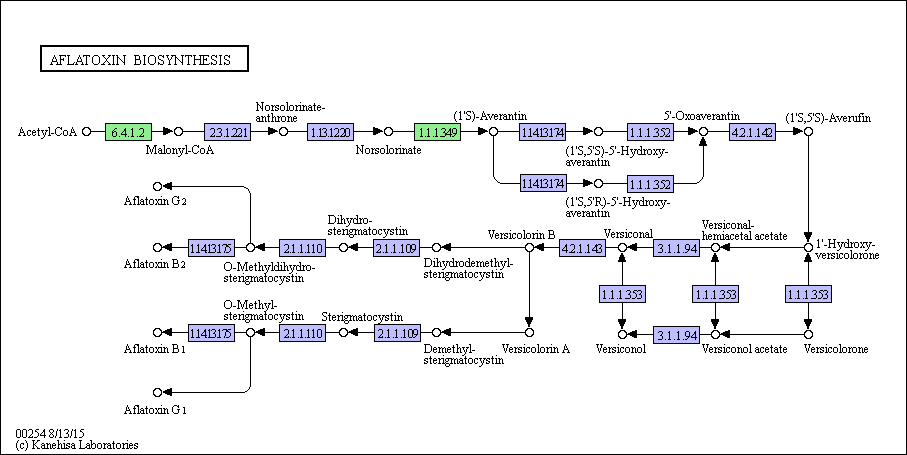
**

**(b)**

**
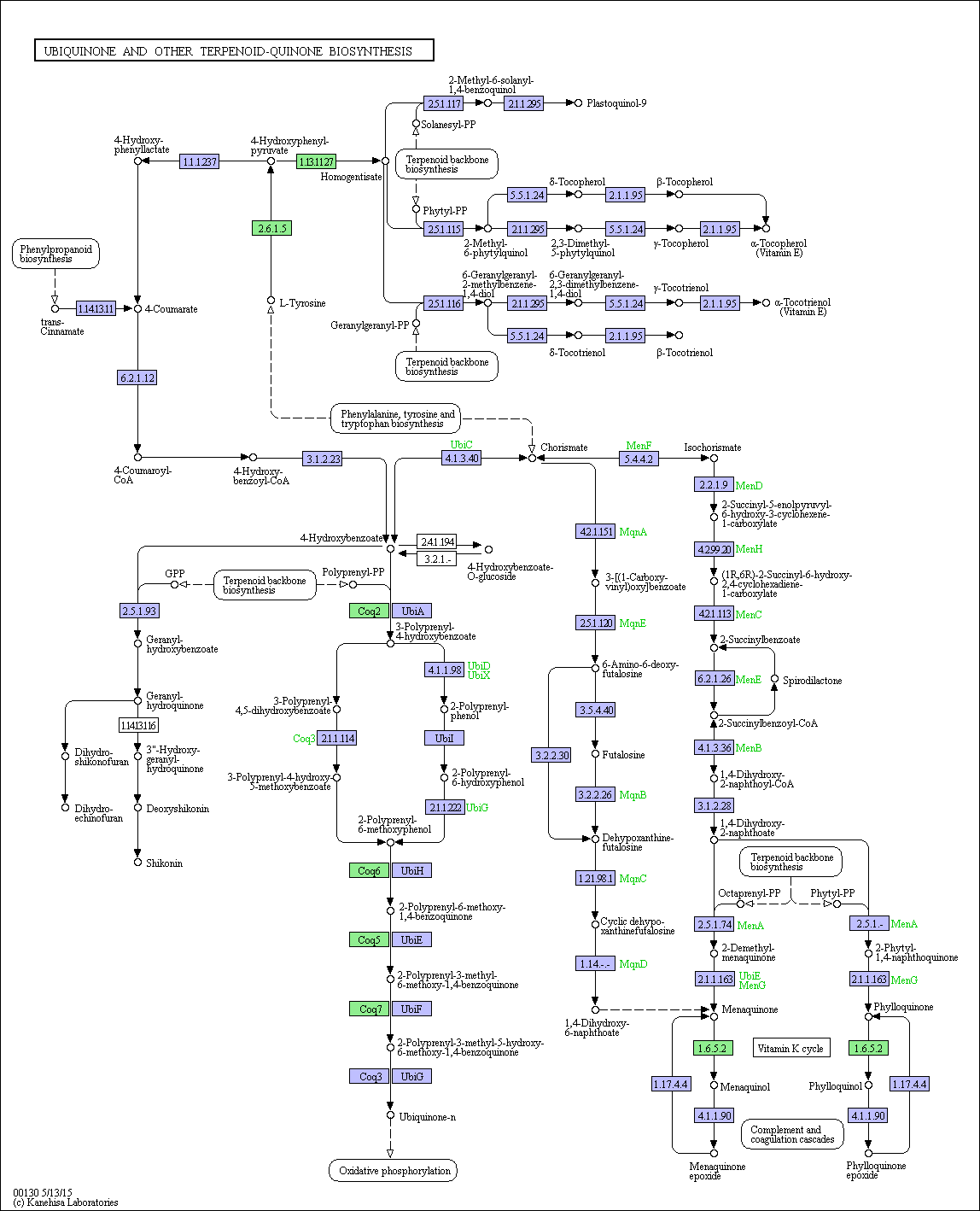
**

**Supplementary Fig. S3.** Aflatoxin and Terpennoids metabolic pathways

**a** Aflatoxim metabolism pathway. **b** Ubiqinone and other terpenoid-ubiquinone biosynthesis pathway.

**
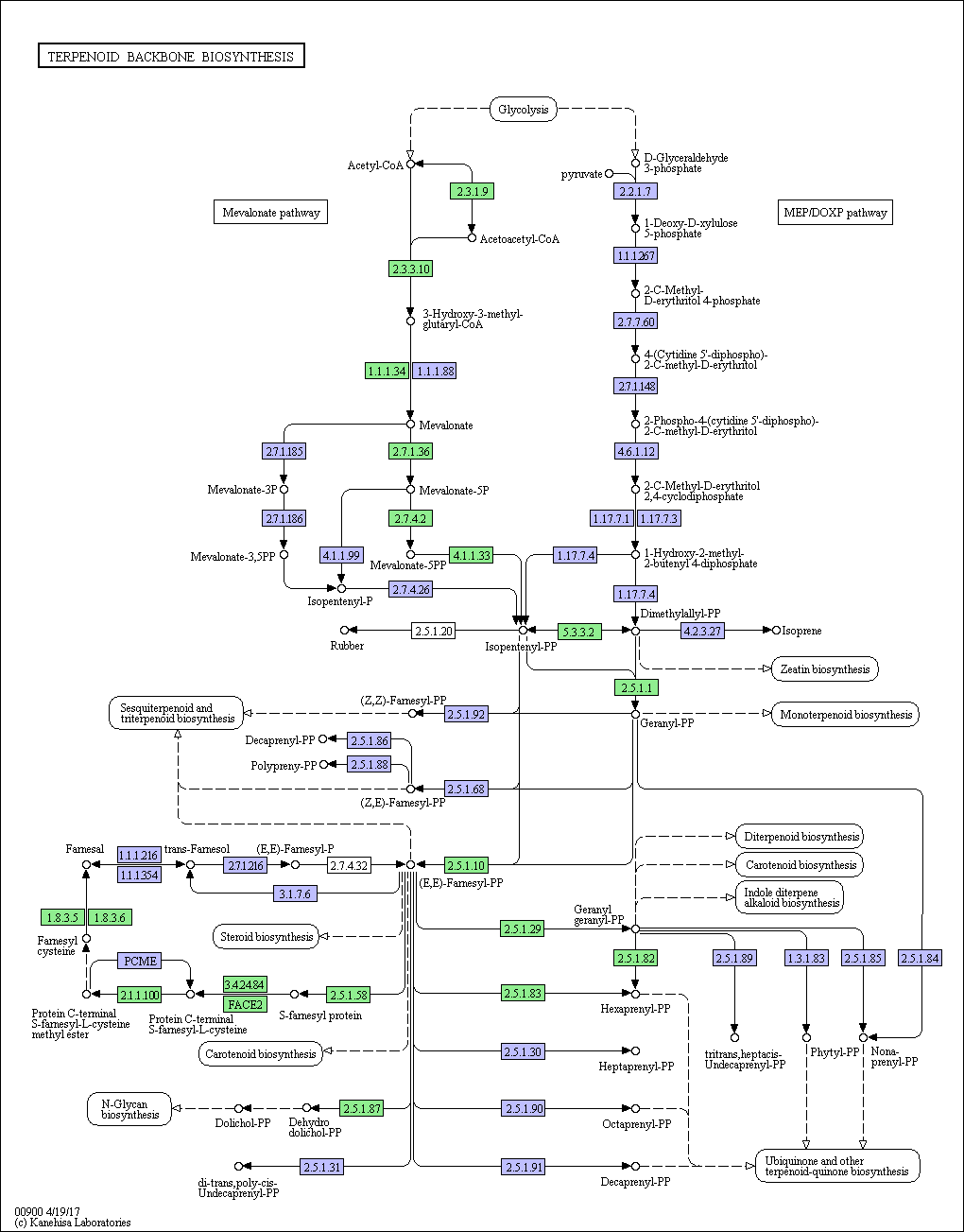
**

**Supplementary Fig. S4.** Terpenoid backbone biosynthesis pathway showing the mevalonate and MEP pathways

**(a)**

**
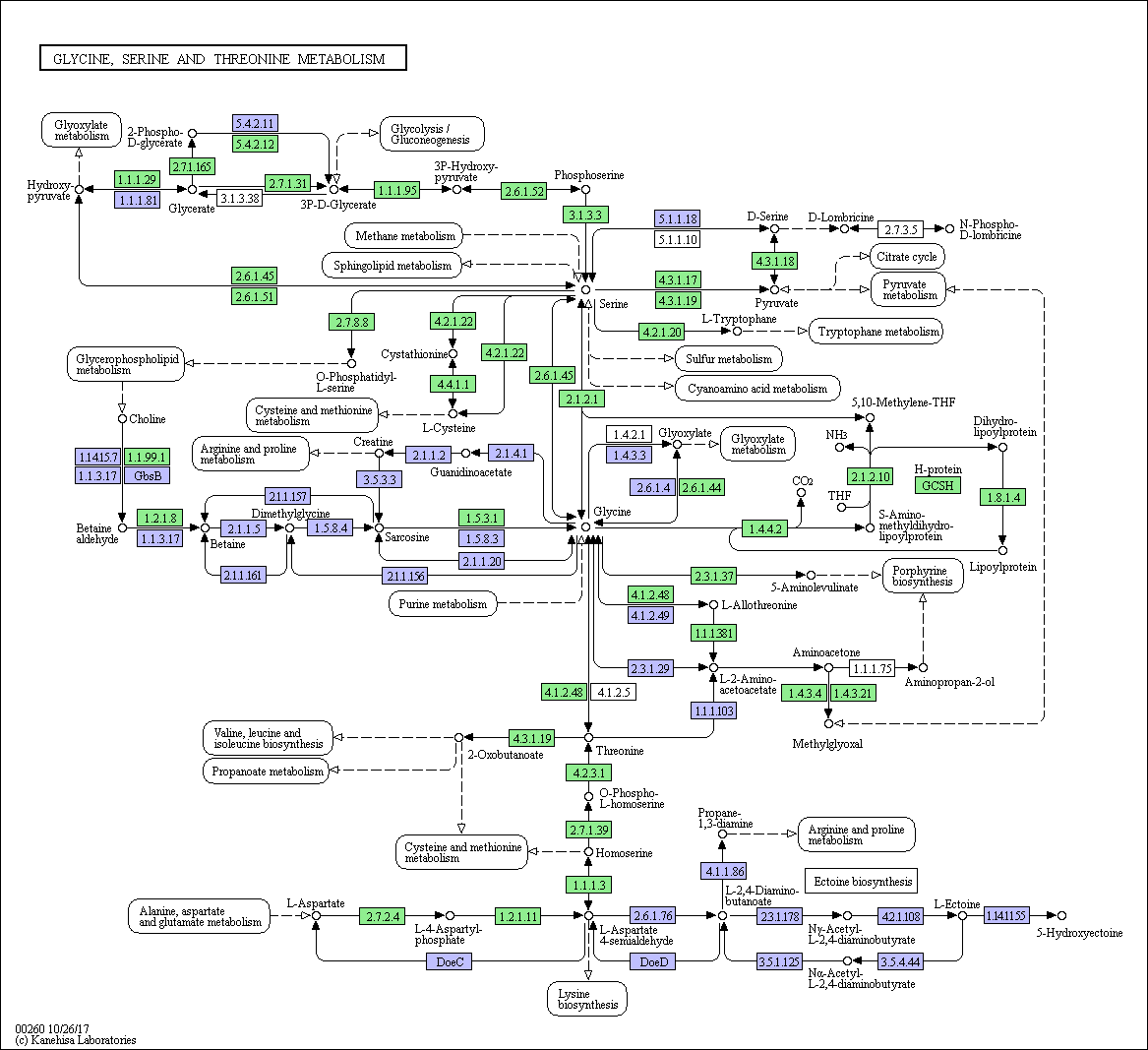
**

**(b)**

**
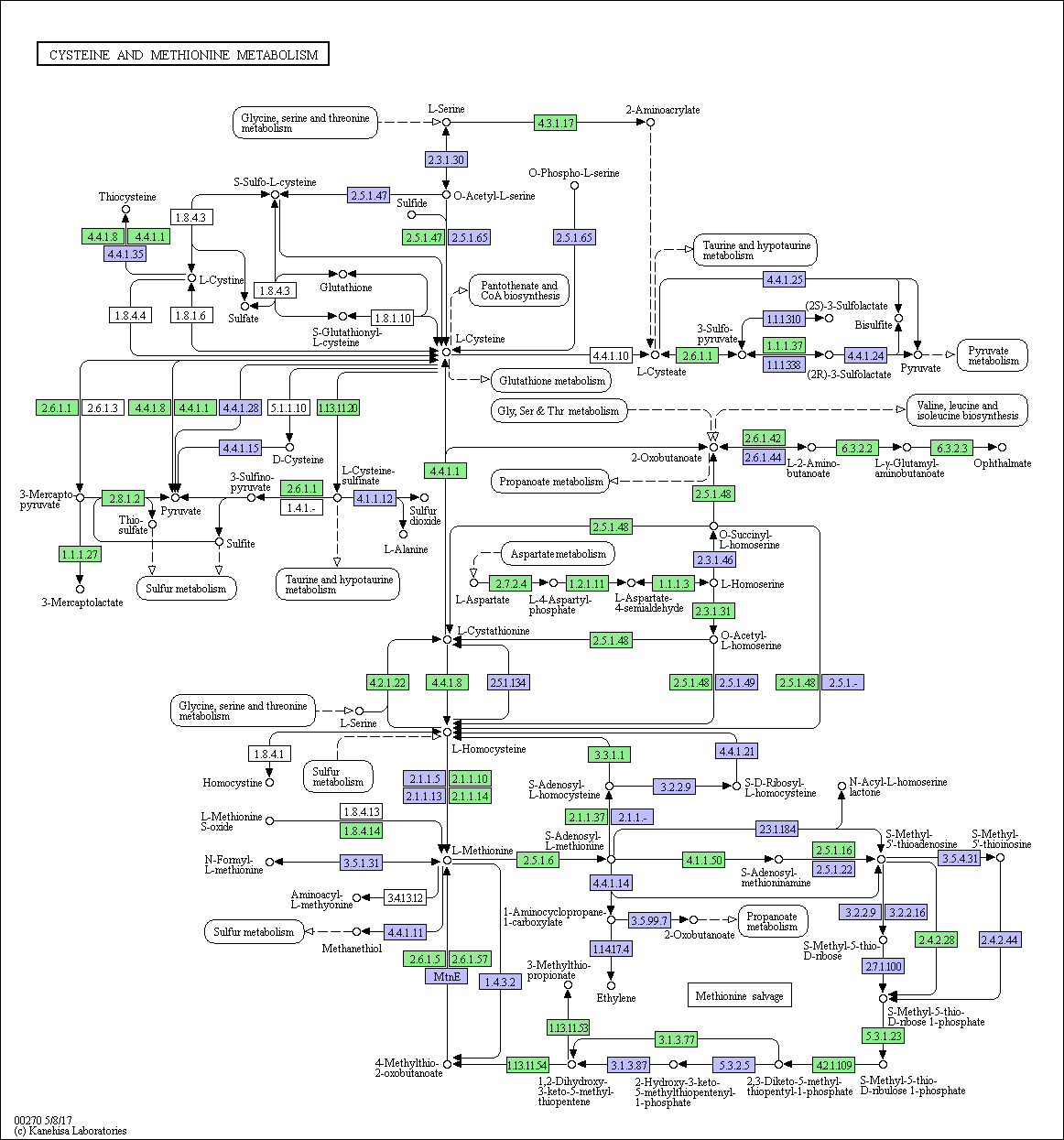
**

**Supplementary Fig. S5.** Metabolic pathways of glycine, serine, and threonine; and cysteine and methionine pathway

**a** Glycine, serine, and threonine metabolism. **b** Cysteine and methionine metabolism.
